# YJMob100K: City-scale and longitudinal dataset of anonymized human mobility trajectories

**DOI:** 10.1038/s41597-024-03237-9

**Published:** 2024-04-18

**Authors:** Takahiro Yabe, Kota Tsubouchi, Toru Shimizu, Yoshihide Sekimoto, Kaoru Sezaki, Esteban Moro, Alex Pentland

**Affiliations:** 1 Institute for Data, Systems, and Society (IDSS), Massachusetts Institute of Technology, Cambridge, MA 02139 USA; 2 Center for Urban Science and Progress (CUSP) and Department of Technology Management and Innovation, Tandon School of Engineering, New York University, Brooklyn, NY 11201 USA; 3 LY Corporation, Kioicho, Tokyo, 102-8282 Japan; 4 Center for Spatial Information Science, The University of Tokyo, Kashiwa, Chiba, 277-8568 Japan; 5 Grupo Interdisciplinar de Sistemas Complejos (GISC), Departamento de Matemáticas, Universidad Carlos III de Madrid, Leganés, 28911 Madrid, Spain; 6 Network Science Institute, Northeastern University, Boston, Massachusetts 02115 US; 7 Media Lab, Massachusetts Institute of Technology, Cambridge, MA 02139 USA

**Keywords:** Social sciences, Geography

## Abstract

Modeling and predicting human mobility trajectories in urban areas is an essential task for various applications including transportation modeling, disaster management, and urban planning. The recent availability of large-scale human movement data collected from mobile devices has enabled the development of complex human mobility prediction models. However, human mobility prediction methods are often trained and tested on different datasets, due to the lack of open-source large-scale human mobility datasets amid privacy concerns, posing a challenge towards conducting transparent performance comparisons between methods. To this end, we created an open-source, anonymized, metropolitan scale, and longitudinal (75 days) dataset of 100,000 individuals’ human mobility trajectories, using mobile phone location data provided by Yahoo Japan Corporation (currently renamed to LY Corporation), named YJMob100K. The location pings are spatially and temporally discretized, and the metropolitan area is undisclosed to protect users’ privacy. The 90-day period is composed of 75 days of business-as-usual and 15 days during an emergency, to test human mobility predictability during both normal and anomalous situations.

## Background & Summary

Understanding, modeling, and predicting human mobility trajectories in urban areas is an essential task for various domains and applications, including human behavior analysis^1^, transportation and activity analysis^2^, disaster risk management^3^, epidemic modeling^4^, and urban planning^5^. Traditionally, travel surveys and census data have been utilized as the main sources of data to understand such macroscopic urban dynamics^6^. The recent availability of large-scale human movement and behavior data collected from (often millions of) mobile devices and social media platforms^7^ have enabled the development and testing of complex human mobility models, resulting in a plethora of proposed methods for the prediction of human mobility traces^8^.

Despite its academic popularity and societal impact, human mobility modeling and prediction methods are often trained and tested on different proprietary datasets, due to the lack of open-source and large-scale human mobility datasets amid privacy concerns^9^. This makes it difficult to conduct fair performance comparisons across different methods. Several efforts have created open-source datasets of human mobility. Real-world trajectory datasets include the GeoLife dataset, T-Drive trajectory dataset, and NYC Taxi and Limousine Commission dataset. The GeoLife dataset^10^ provides trajectory data of 182 users across a period of over three years, containing 17,621 trajectories with a total distance of about 1.2 million kilometers and a total duration of 48,000 hours. The T-Drive trajectory dataset contains trajectories of 10,357 taxis across a one-week timeframe^11^. The total number of points in this dataset is about 15 million and the total distance of the trajectories reaches 9 million kilometers. Similarly, the New York City Taxi and Limousine Commission (NYC-TLC) provides pick-up and drop-off locations and timestamps data^12^. Although T-Drive and NYC-TLC datasets provide massive amounts of trajectory information, they are limited to taxi trips. Moreover, during the COVID-19 pandemic, several human mobility datasets have been published to analyze the impacts of lockdown policies on human behavior, including aggregate origin-destination matrices^13,14^ and network based indexes that describe the potential encounters across regions^15^. These mobility metrics can be combined with statistics about the pandemic spread^16^ to draw conclusions on the effectiveness of various policies.

While such datasets are valuable in conducting large-scale experiments on human mobility prediction, the lack of metropolitan-scale, longitudinal, real-world, and open-source datasets of individuals has been one of the key barriers hindering the progress of human mobility model development. Lack of metropolitan-scale and longitudinal data limits human mobility researchers from developing computational models that capture and predict the general dynamics of urban mobility patterns across regions. While synthetic datasets produced from open-source data, including the Open PFLOW^17^ and Pseudo-PFLOW datasets^18^, have become more available, studies have found that such models struggle to produce meaningful sequences of geo-locations with reasonable trip lengths and to model traffic flow at intersections accurately^19^.

To this end, we created an open-source and anonymized dataset of human mobility trajectories from mobile phone location data provided by Yahoo Japan Corporation (now called LY Corporation). The dataset contains 100,000 individuals’ mobility trajectories across a 75 day period collected from an undisclosed, highly populated metropolitan area in Japan. The location pings are discretized into 500 meters × 500 meters grid cells and the timestamps are rounded up into 30-minute bins. The actual date of the observations is not available either (i.e., timeslot *t* of day *d*) to protect privacy. In the second Dataset, the 75 day period is composed of 60 days of business-as-usual and 15 days during an emergency with unusual behavior.

To promote the use of the dataset, we hosted a human mobility prediction data challenge (‘HuMob Challenge 2023’) using the YJMob100K dataset^20^. The workshop was held in conjunction with ACM SIGSPATIAL 2023^21^, and more than 20 submissions for human mobility prediction methods were submitted, and the 10 top performing methods were presented and published. These studies provide various examples of use cases of this dataset^22^.

## Methods

### Observation of smartphone GPS records

**Fig. 1:**
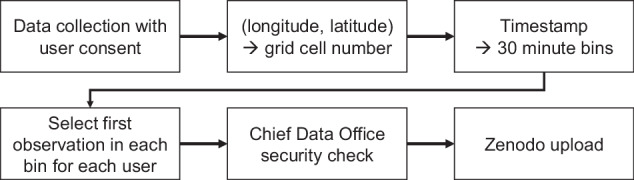
Diagram showing the logistics of data collection, processing, and sharing.

The mobile phone location data was collected, processed, and shared through the workflow shown in Fig. 1. GPS location data were collected from smartphones that have installed Yahoo Japan (currently called LY) applications, via author affiliation (LY Corporation). The data points were anonymized so that individuals cannot be specified, and personal information such as gender, age, and occupation are unknown. LY Corporation explicitly states that the anonymized data will be used for research and analysis with research partners, in the privacy policy section 5e,which the users have agreed with when installing the smartphone application^23^.

Since the dataset is de-identified and anonymized through the redaction of the actual date and location coordinates, the Human Research Protection Program in the Institutional Review Board (IRB) at New York University determined that the data does not meet the federal regulations definition of human subject, and therefore, it is not under the purview of the IRB.

Each GPS location record contains the user’s unique ID, timestamp of the observation, longitude, and latitude, and the data has a sample rate of approximately 5% of the entire population living within the grid cell area, according to the national census data obtained from the National Land Information Division, National Spatial Planning and Regional Policy Bureau, Ministry of Land, Infrastructure, and Transport of Japan^24^. The data acquisition frequency of GPS locations varies according to the movement speed of the user to minimize the burden on the user’s smartphone battery. If it is determined that the user is staying in a certain place for a long time, data is acquired at a relatively low frequency, and if it is determined that the user is moving, the data is acquired more frequently.

### Spatio-temporal processing and anonymization

**Fig. 2:**
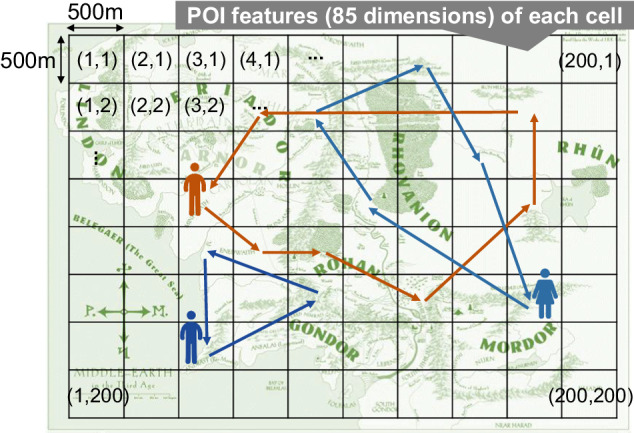
Human mobility trajectories are discretized into 500 meters × 500 meter cells inside a target area that spans 200 × 200 grid cells, and into 30 minute intervals. The city where the data was observed, nor the exact date and time of the observations are hidden to protect user privacy.

As shown in Fig. 2, the set of mobile phone users included in the dataset was selected by spatially and temporally cropping the raw dataset. To spatially crop the raw dataset, we created a boundary box around an undisclosed metropolitan area in Japan and selected mobile phone users who were observed within the boundary box more than 10 times during a 10-day period (dates undisclosed for privacy reasons). To make the mobile phone users unidentifiable, the location pings are discretized into 500 meters × 500 meters grid cells and the timestamps into 30-minute bins. The actual date of the observations was also masked (i.e., timeslot *t* of day *d*) to protect privacy. The movement (encoded into 500 m grid cells) of the mobile phone users was tracked across a total of 75 days (again, dates are undisclosed). *Dataset 1* includes a 75-day period of business-as-usual period, while *Dataset 2* contains 60 days of business-as-usual period and a 15-day period during an emergency situation, where we can assume human behavior and mobility patterns could be shifted. The dataset was finally cropped by selecting users with a sufficient number of 30-minute timeslot observations to ensure that the mobility patterns could be studied. Observations outside of the target boundary box were discarded. For *Dataset 1*, 100,000 users were selected, and for *Dataset 2*, 25,000 users were selected.

### Privacy policy

Yahoo Japan Corporation (renamed to LY Corporation on October 2023) has developed its privacy policy and requires users to read and agree to its privacy policy before using any of the services provided by Yahoo Japan (LY Corporation). Furthermore, because location data is highly sensitive for the users, users were asked to sign an additional consent form specific to the collection and usage of location data when they used apps that collect location information. The additional consent explains the frequency and accuracy of location information collection, and also the purpose and how the data will be used. Moreover, strict restrictions were implemented in the analysis procedure. The methodology for handling the data and for obtaining user consent for this study was supervised by an advisory board composed of external experts. It was also ensured that external research institutions that participate in this study (including co-investigators) do not have direct access to the data. Although external research institutions were allowed to analyze aggregated data, the actual raw data were kept within the internal company servers, and any analysis performed on raw data was performed within servers administered by the company.

## Data Records

### Provided datasets

The YJMob100K dataset is available at Zenodo^25^. The YJMob100K dataset consists of 4 datasets–human mobility datasets #1 and #2 (which are derived from the original human mobility dataset), the POI dataset which may be used to supplement the prediction of human mobility, and the list of POI categories. The entire dataset consists of the following four datasets:

- Human mobility datasets
  - YJMob100K human mobility dataset #1 (yjmob100k-dataset1.csv.gz)
  - YJMob100K human mobility dataset #2 (yjmob100k-dataset2.csv.gz)

- POI dataset (cell_POIcat.csv.gz)
- POI category list (POI_datacategories.csv)

### Human mobility datasets

The human mobility datasets contain the movement of individuals during a 75 day period. Table 1 shows an example of the dataset provided. In both human mobility datasets, each record refers to an observation of an individual which consists of the following columns:

- user ID is the unique identifier of the mobile phone user (type: integer)
- day is the masked date of the observation. It may take a value between 0 and 74 for both Dataset 1 and Dataset 2 (type: integer).
- timeslot is the timestamp of the observation discretized into 30 minute intervals. It may take a value between 0 and 47, where 0 indicates between 0AM and 0:30AM, and 13 would indicate the timeslot between 6:30AM and 7:00AM.
- x,y are the coordinates of the observed location mapped onto the 500 meter discretized grid cell. It may take a value between (1, 1) and (200, 200). Details are shown in Fig. 2.

**Table 1:** Example of dataframe and the columns in the human mobility trajectory datasets.

user ID (String)	day (Integer)	timeslot (Integer)	x (Integer)	y (Integer)
1	1	13	10	13
1	1	18	11	15
1	1	24	11	17
1	1	27	12	19
		…		
2	3	15	31	19
2	3	28	35	33
2	4	12	35	36
		…		

*Dataset 1* contains the individual movements of 100,000 individuals during a 75-day business-as-usual scenario. *Dataset 2* contains the individual movements of 25,000 individuals during a 60-day business-as-usual period and a 15-day emergency scenario. Due to anonymization requirements, the nature of the emergency cannot be disclosed, however, the objective is to provide data users to test the generalizability of their prediction algorithms to out-of-sample situations.

### POI dataset

To aid the prediction task, we have prepared an auxiliary dataset that provides the count of different points-of-interest categories in each grid cell as geographical context information (e.g., restaurants, cafes, schools). However, to maintain the anonymity of the location, we are not able to provide the actual category name that corresponds to each dimension. Therefore, each cell has an 85-dimensional vector, as shown in Table 2. The names of the 85 POI categories (e.g., Japanese restaurant, shopping) are provided in the POI category list (POI_datacategories.csv).

**Table 2:** Example of dataframe and the columns in the POI category dataset.

x (Integer)	y (Integer)	POI category (Integer)	# of POIs (Integer)
1	1	13	10
1	1	18	11
1	1	24	11
1	1	27	12
		…	
2	2	15	31
2	2	28	35
2	2	12	35
		…	

First two columns show the x and y coordinates of the grid cell, third column denotes the dimension of the POI category (between 1 and 85), and the fourth column shows how many POIs of the POI category dimension located in the grid cell.

## Technical Validation

### Correlation with census population data

**Fig. 3:**
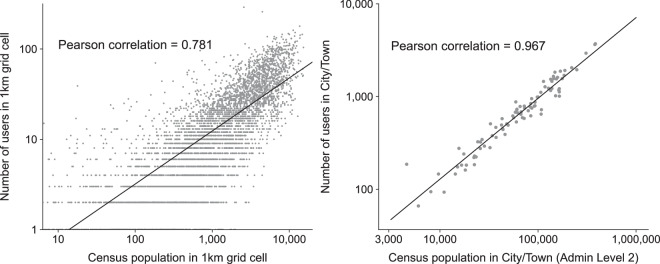
Validation of the human mobility data. (left) Correlation plot between the census population in each 1 km grid cell (x-axis) and the number of user IDs who reside in each 1 km grid in the YJMob100K dataset. The Pearson correlation is 0.781, and shows high agreement with census data, showcasing the spatial representativeness of the data. (right) On the city and town level (administrative boundary level 2), the correlation between the mobility data and census population is extremely high, with a Pearson correlation of 0.967.

The spatial distribution of the 100,000 individuals was further validated using census population data. The census data was obtained from the National Land Information Division, National Spatial Planning and Regional Policy Bureau, Ministry of Land, Infrastructure, and Transport of Japan^24^. Each individual’s home location was estimated by taking the most frequently visited cell between 8 PM (timeslot 40) and 8 AM (timeslot 16). The number of individuals in the YJMob100K dataset were grouped into 1 km grid cells by their estimated residential locations. Figure 3 shows the correlation plot between the census population in each 1 km grid cell (x-axis) and the number of user IDs who reside in each 1 km grid in the YJMob100K dataset. The Pearson correlation is 0.796, and shows high agreement with census data, showcasing the spatial representativeness of the data. On the city and town level (administrative boundary level 2), the correlation between the mobility data and census population is extremely high, with a Pearson correlation of 0.967.

## Usage Notes

### Statistics of the data

**Fig. 4:**
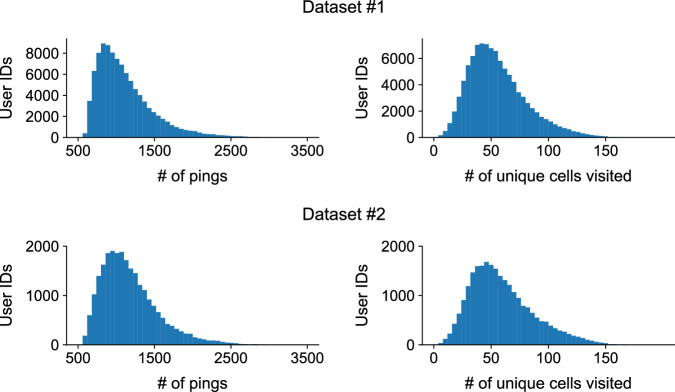
Histograms of the number of GPS location data pings and number of unique cells visited per user, across the 75 day period stored in Dataset 1.

**Fig. 5:**
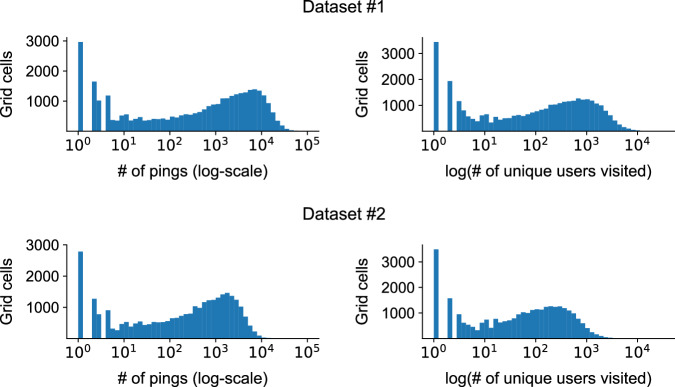
Histograms of the number of GPS location data pings and number of unique users visited per grid cell, across the 75 day period stored in Dataset 1.

To provide guidance for data users, we have computed the basic descriptive statistics of both *Dataset 1* and *Dataset 2*. In *Dataset 1*, the total number of records are 111,535,175, with exactly 100,000 unique users (numbered 0 to 99, 999), across 75 days (numbered 0 to 74), in 48 different 30 minute timesteps (numbered 0 to 47). *Dataset 2* contained a total of 29,389,749 records. Figure 4 shows the histogram of the number of pings per user ID (left) and the number of unique cells visited per user ID (right). All plots show a skewed distribution, where a small fraction of the users are observed many times (i.e., more than 2000 pings, at 100 unique cells). Figure 5 shows the histogram of the number of pings per user ID (left) and the number of unique users visited to each grid cell (right). Note that the x-axis in both plots are log-scaled. Both plots show a bimodal distribution, where a large fraction of the cells are visited very few times (less than 10 pings or unique users) while another mode can be observed at around 10000 pings and 1000 unique users visited. This highlights the mix of urban and rural areas in the target region.

**Fig. 6:**
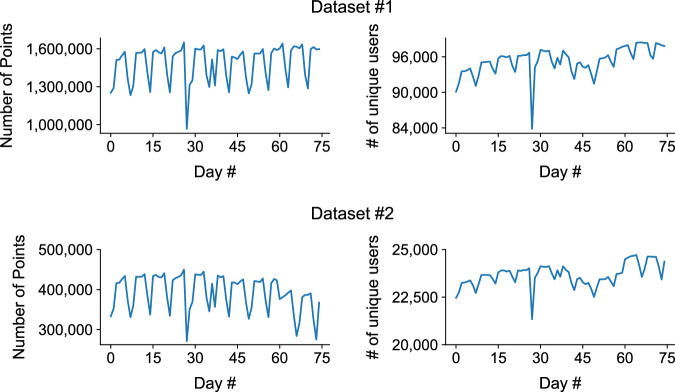
Temporal dynamics of the number of pings and unique users per day (from day 0 to 74) in Dataset 1.

**Fig. 7:**
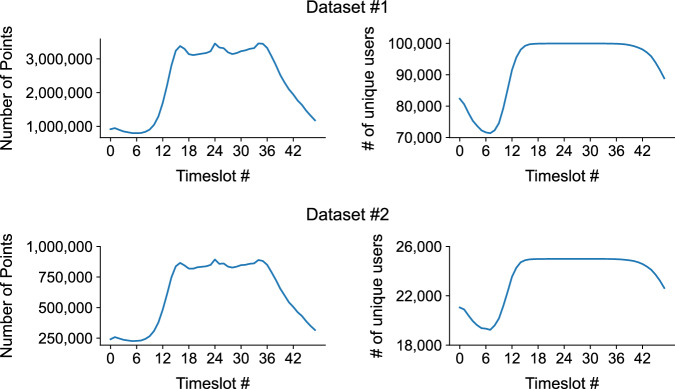
Temporal dynamics of the number of pings and unique users per timeslot (from timeslot 0 to 47) in Dataset 1.

**Fig. 8:**
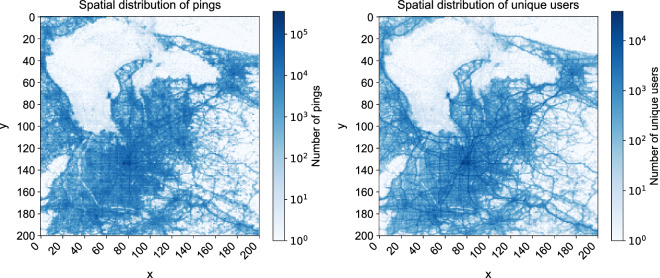
2-dimensional histogram of the number of pings and the number of observed unique users across the 75 days. Note that the scales are log-scaled. The patterns show clear urban (blue) and rural (red) areas.

Figure 6 shows the temporal dynamics of the number of pings and unique users per day (from day 0 to 74) in Dataset 1. The patterns show temporal regularity, showing clear patterns of weekdays and weekends. There is an anomaly on day 27, however this is due to a data collection issue. The unique number of users observed each day fluctuates more, showing a decrease near days 40 to 50 and an increase from day 60 onwards. We have noticed that there is a decrease in the number of points and users on day 27 in both datasets. This was due to a natural disaster that occurred in the area. Due to the anonymity of the location, we are not able to disclose the nature of the event. We advise data users to exclude day 27 from the analysis. Figure 7 shows the temporal dynamics of the number of pings and unique users per timeslot (from timeslot 0 to 47) aggregated across all days observed in Dataset 1. The patterns show temporal regularity, showing clear morning and daytime peaks. The unique number of users observed between timeslot 12 (6AM) and timeslot 40 (8PM) is stable at around 100,000, showing a high observability during those time periods. Figure 8 shows a 2-dimensional histogram of the number of pings and the number of observed unique users across the 75 days. Note that the scales are log-scaled. The patterns show clear urban (blue) and rural (red) areas.

### Limitations of the data

As with any dataset, the YJMob100K dataset should be used in light of several limitations. First, to enable the sharing of the dataset with an unprecedented size while preserving the privacy of individual users, we were required to anonymize several aspects of the data, including the name of the city, actual longitude and latitude values of each grid cell, and the actual POI information. Therefore, this dataset should not be used for understanding the social dynamics at the POI scale, such as the analysis of social segregation^26^. Second, due to the extensive anonymization, individual characteristics of users (e.g., home locations, work locations) are not provided. Typically, individual users’ sociodemographic and economic characteristics are inferred using the estimated home location, however, due to the anonymization of the city or the exact longitude and latitude information, that is not possible. The YJMob100K dataset is intended to serve the scientific community as a benchmark data for human mobility prediction tasks, not for urban science or human behavioral studies.

## Data Availability

No custom code was generated for this work.
